# Reimagining digital healthcare with a patient-centric approach: The role of user experience (UX) research

**DOI:** 10.3389/fdgth.2022.899976

**Published:** 2022-08-09

**Authors:** Rose M. Gilbert

**Affiliations:** ^1^ Ophthalmology Department, Addenbrookes Hospital, Cambridge University Hospitals NHS Foundation Trust & Moorfields Eye Hospital NHS Foundation Trust, London, United Kingdom; ^2^ NIHR Moorfields Biomedical Research Centre, London, United Kingdom

**Keywords:** digital, healhcare, patient centric, user research, Amazon, customer focus, patient involvement, innovation

## Introduction

Central to the United Kingdom's (UK) National Health Service (NHS) Long Term Plan (1), setting out the UK NHS' ambitions for improvement over the next decade, is the focus on technology in the future NHS. The plan sets out “critical priorities” that will support digital transformation and provision of health and social care in the UK. Specific aims include “straightforward digital access to NHS services” with a focus on “empowering people” by “the ability to access, manage and contribute to digital tools, information and services”.

Digital health interventions ultimately aim to improve health services and the health and quality of life of patients, but often, the involvement of the patients, themselves, is missed. In other sectors, when a new product is being brought to the market, consumers are involved in the design process at an early stage. It seems logical to do this because they are, in fact, the people who will use the product. Having the product users involved is likely to ensure the product is easy to use and fit for purpose, so that they will purchase, and re-purchase, it.

Jeff Bezos’ letter to shareholders from Amazon's 1997 Annual Report (2), is still relevant today for its emphasis on customer outcomes, specifically the idea that long term success would stem from continuing to “relentlessly focus” on customers. Amazon works to generate customer loyalty by focussing on engagement, conversion and satisfaction. Mastery of the “purchase and repeat purchase” feedback loop using customer-centric methods has ultimately determined Amazon's market success. Healthcare systems have been slow to adopt this approach. It might seem obvious that there is a need for involving the end users (whether they are health professionals, patients, or both) in the design process from the early stages in order to enable their needs and characteristics to be identified (3), however, this is not often observed in practice. If patients are involved in development of digital health products or services, this is most often in the final stages of a project, for example, *via* usability testing (4) to evaluate the product or service. However, involving patients in the early stages of design of a digital health intervention is crucial to finding patient-focussed solutions. Ideally, they would be incorporated as equal partners the design phase, that is “co-production”, providing active input rather than being passive recipients of services (5). Co-production emphasises that the people who use services have assets which can help to improve those services, rather than simply needs which must be met (5). Patients (and often their carers) have “lived experiences” of disease and intimately know the day-to-day difficulties of functioning with a particular condition (6). This data needs to be captured and fed into product design. Clinician experts also need to be involved and most often will make the final decisions on products, but the development process should ideally involve the public and patients at all stages.

## User experience (UX) research in healthcare

User research (7) is a key part of user-centric design, because when you learn about users of your proposed services, it facilitates creating services that meet their needs. User experience (UX) research is the systematic study of target users and their requirements, to add realistic contexts and insights to design processes. The success or failure of a digital health innovation often depends on how it is received by the user. In some cases, the user might be making adjustments or adaptions to the context or the product to make it work (invisible work), which needs to be observed and understood in order for successful integration of a product or service to occur (8). Healthcare needs UX researchers who understand the relevant patient population, the clinicians and, also, the system in which the product will operate. Current efforts to create, study, and disseminate digital health have been limited by lack of user engagement in the design process (9) and stands to reason that the ability to engage with the target patient population is a prerequisite to successful UX research.

Various processes and methodologies for UX research in healthcare have been described in the literature (10–12), however, qualitative methods such as focus and/or discussion groups are a common component of these. An integrative review of published qualitative methods of user experience research (all deemed successful in setting up health apps) has proposed a structure of four sessions, in which information technology and health professionals and patients take part (3). These sessions are summarised as follows: composing, preparing, and organizing contents (session 1); testing structure and usability (session 2); does the app fit the needs of end users? (session 3); and last, testing-keep on improving (session 4). Following the initial focus group discussions for situation analysis and information architecture, another study described a user-centred design process for developing an mHealth app incorporating further design sessions (design activity 1 for wireframe designing, design activity 2 for wireframe testing) followed by user testing (sessions 1 and 2) (12). A study to develop a patient-centred health platform and data repository described a high-level schematic work-flow cycle comprising of the following four steps: 1. Research, 2. Design, 3. Development and 4. Evaluate and Iterate, which engaged patients using both qualitative methods (interviews, focus groups and interactive workshops) and quantitative methods (survey) throughout the process (13).

Design science research, itself, has been identified as a unique research paradigm, which can be analysed as having three defined, interrelated, cycles: the Relevance Cycle, the Rigor Cycle and the Design Cycle (14). This has been developed into a theoretical framework: the Information System Research (ISR) framework and has been used to guide the implementation of user-centred human–computer interaction research methods to identify mHealth needs of users, mobile app design preferences; and the barriers and facilitators that prohibit or encourage the uptake and sustained use of mobile apps (10). In the Relevance cycle, focus groups were conducted with targeted end-users. In the Rigor cycle, a review was undertaken to identify technology-based interventions for meeting the health prevention needs of the target population. In the Design Cycle, usability evaluation methods were employed to iteratively develop and refine mock-ups for a mHealth app. In summary, there are a range of methodologies and frameworks which can be used by UX researchers to uncover problems and design opportunities within the healthcare field under study.

## Conclusion

UX research is a central tenet in the process of developing user-centric products and services. It requires specialised researchers with a range of skills and experience including digital healthcare literacy, health psychology (including qualitative and quantitative methods), and ideally domain experience in the clinical area in which the technology is being developed. Too often, digital development in large healthcare organisations shifts its focus from people to process. Our challenge in healthcare is to understand how to create feedback loops that ensure digital health services are genuinely patient-centric, as opposed to development processes serving the needs of the teams creating them. Barriers to patient involvement can be overcome if we take Jeff Bezos' approach of “obsessing” over our customers: the patients.
